# A Survey of the Common Mutations and IVS8-Tn Polymorphism of Cystic Fibrosis Transmembrane Conductance Regulator Gene in Infertile Men with Nonobstructive Azoospermia and CBAVD in Iranian Population

**DOI:** 10.29252/.23.2.92

**Published:** 2019-03

**Authors:** Fatemeh Asadi, Reza Mirfakhraie, Farzaneh Mirzajani, Azam Khedri

**Affiliations:** 1 Department of Molecular Genetics, Marvdasht Branch, Islamic Azad University, Marvdasht, Iran;; 2 Department of Molecular Genetics, Science and Research Branch, Islamic Azad University, Fars, Iran;; 3 Department of Medical Genetics, Shahid Beheshti University of Medical Sciences, Tehran, Iran;; 4 St. Justine Hospital, Montreal University, Montreal, Canada;; 5 Medical Genetics Department of the National Institute of Genetic Engineering and Biotechnology, Tehran, Iran;; 6 Department of Biochemistry, Faculty of Medicine, Tehran University of Medical Science ,Tehran, Iran

**Keywords:** Azoospermia, CFTR, Congenital bilateral absence of the vas deferens, Male Infertility, Mutation

## Abstract

**Background:**

Studies have revealed a strong association between mutations of *CFTR* gene and the congenital bilateral absence of the vas deferens (CBAVD), but the role of this gene in other types of male infertility is still unclear. The purpose of this study was to *investigate* the frequency of the most common mutations of the *CFTR* gene (F508, G542X, N1303K, G551D, and W1282X) in a population of infertile men with nonobstructive azoospermia (NOA) and CBAVD in Iran.

**Methods:**

Blood samples were obtained from 50 NOA, 50 CBAVD, and 100 normal males (control). Genomic DNA was isolated from whole blood leukocytes, and the presence of common mutations of the *CFTR* gene was assessed by an amplification refractory mutation system-polymerase chain reaction (ARMS-PCR). Restriction fragment length polymorphism (PCR-RFLP) was also used to analyze IVS8-Tn polymorphism.

**Results:**

It was found that 16%, 8%, and 8% of *patients with CBAVD* were heterozygote for F508, G542X, and N1303K, respectively. The frequency of the 5T allele was 34% and *higher than the normal group* (*p* < 0.001). None of the common *CFTR* gene mutations were detected in NOA patients, and no *significant *difference was found in the distribution of the 5T *allele* between the NOA patients and the control group (5 vs. 3 *p* = 0.721).

**Conclusion:**

Based on the present case-control study, the *CFTR* gene mutations and IVS8-Tn polymorphisms are correlated with CBAVD; however, extensive investigations are necessary to determine the exact relationship between the gene mutations and other forms of male infertility.

## INTRODUCTION

Infertility is commonly known as the inability to become pregnant after one year of frequent intercourse, in the absence of a contraceptive^[^^1^^]^. Male infertility is a major health problem in the global populations; its prevalence in Western countries has been estimated to be about 20%^[^^2^^]^. The main causes of male factor infertility are azoospermia, asthenozoospermia, teratozoospermia, and oligo-zoospermia. Azoospermia is characterized as the lack of sperms in the ejaculate, and it can be divided into two groups: nonobstructive azoospermia (NOA) and obstructive azoospermia (OA). The former is usually *due to spermatogenesis failure, while *the latter is caused by an obstruction *in* the seminal tract (epididymis, vas deferens, and ejaculatory ducts)^[^^3^^-^^5^^]^. The congenital bilateral absence of the vas deferens (CBAVD) accounted for 25% of cases of OA and 1.5% of male infertility^[^^6^^,^^7^^]^. 

 It has been estimated that more than 3000 genes are involved in the genetic regulation of male or female fertility^[^^8^^-^^10^^]^. The *CFTR *gene is located on chromosome 7 (7q31.2) and contains 27 exons that span 190 kb of genomic DNA. It encodes a chloride channel in epithelial cells. Mutations in this gene result in cystic fibrosis (CF). The clinical manifestations of CF include chronic obstructive pulmonary disease, pancreatic insufficiency, high levels of sweat electrolytes, and male infertility^[^^11^^,^^12^^]^. In most cases, CBAVD is regarded as a genital form of CF, without other clinical manifestations^[^^13^^]^. More than 2000 mutations of the *CFTR *gene have been found, which has led to a variety of clinical phenotypes of CF^[^^14^^]^. F508, N1303K, and G542X have been identified as the most frequent *CF*-*causing mutations* worldwide. Several studies have reported the role of *CFTR* gene mutations in CBAVD in men and congenital unilateral absence of the vas deferens^[^^11^^,^^15^^-^^17^^]^. The *CFTR *gene mutations associated with this disorder are as follows: F508, R117H, G551D, D1152H, G542X, M470W, R334W, R74W, M9521, W1282X,  N1303K, and  G85E^[^^18^^-^^20^^]^. *Based on the phenotypic effects*, there are usually two types of alleles in the CF genotype: (1) severe allele and (2) mild allele. F508 and G542X are characterized as severe alleles, while R117H is regarded as a mild allele. Numerous reports have indicated that *CFTR* gene can be involved in another cases of infertility other than CBAVD^[^^21^^-^^23^^]^. However, it is not known for sure whether these mutations have *an important function *in the *spermatogenesis and *NOA.

The present study aimed to evaluate the frequency of common mutations of the CFTR gene including, F508, G551D, G542X, N1303K, and W1282X in Iranian infertile men with NOA and CBAVD using ARMS-PCR technique. Also, IVS8-Tn polymorphism was analyzed by RFLP-PCR.

## MATERIALS AND METHODS

**Fig. 1:**
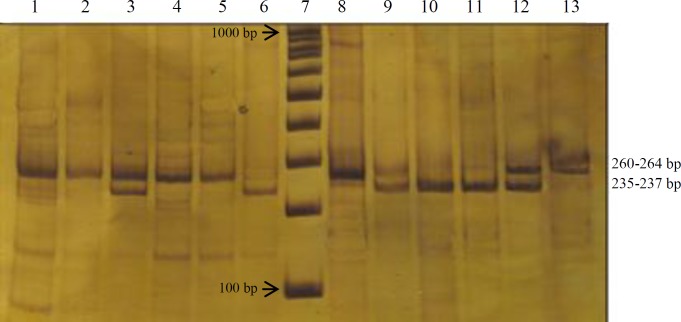
Ag-NO_3_-stained polyacrylamide gel for the evaluation of IVS8-Tn polymorphism. Lanes 1 and 8, *uncut PCR products;* lanes 2-6, digestion results of CF5T/CF9RR products; Lane 7, 100 pb DNA ladder; lanes 9-13, digestion results of CF7T/CF9RR products. Lanes 2 and 9, 7T/9T; lanes 3 and 10, 5T/7T; lanes 4 and 11, 7T/7T; lanes 5 and 12, 7T/9T; lanes 6 and 13, 5T/5T

This case-control study was conducted on 50 CBAVD patients, 50 NOA patients, and a control group (n = 100), who had at least one child; the patients came from Yazd Infertility Center (Yazd) and Mirza Koochak Khan Hospital (Tehran, Iran). The study was conducted at the Medical Genetics Department of the National Institute of Genetic Engineering and Biotechnology (NIGEB, Tehran). The diagnosis of CBAVD was initially suggested by palpable scrotal vas deferens on physical examination and transabdominal/rectal ultrasonography and then confirmed by cytobiochemical characteristics, according to the World Health Organization criteria^[^^24^^,^^25^^]^. The diagnosis of NOA was based on the following examinations: normal semen volume, normal testicular size, presence of the vas deferens by clinical examination, and normal levels of serum follicle-stimulating hormone and also according to the medical history of patients; none of them had chromosomal aberrations or Y-chromosome microdeletions. No other symptoms of CF such as chronic lung inflammation/ infection, pancreatic insufficiency, and intestinal obstruction were observed in these patients. All subjects gave their written informed consent, and then 5 mL of whole blood sample was collected in EDTA vacuum tubes (Becton Dickinson, USA). Genomic DNA was extracted from peripheral blood leukocyte by salting-out method and was analyzed for the most common CF mutations, including F508, G551D, G542X, N1303K, and W1282X using amplification refractory mutation system-polymerase chain reaction (ARMS-PCR) method as described previously^[^^26^^]^. IVS8-Tn polymorphism was analyzed by using restriction fragment length polymorphism (RFLP)-PCR. The CF intron 8/exon 9 (product size: 259-261 bp) primers were as follows: common downstream primer, CF9RR: GACATGGACACCA AATTAAG; upstream primer, CF5T: TGTGTGTGTGTGTGTGTT G*TT), and upstream primer, CF7T: GTGTGTGTGTGTGTGTTTTG*TT; *denotes a mismatch. Amplification reaction was performed using a conventional protocol: the reaction mixture contained 10 mM of Tris-HCl (pH 8.3), 50 mM of KCl, 1.5 mM of MgCl_2_, 0.01 mg/mL of gelatin, 0.2 mM of each deoxynucleotide, and 0.75 mM of each primer and 1 U of AmpliTaq Polymerase in a total volume of 25 µl. The PCR thermal cycles were: 94 °C for 5 min, followed by 32 cycles of 1 min at 94 °C, 1 min at 58 °C (annealing), 1 min at 72 °C (extension), and a final extension of 5 min at 72 °C. Twenty microliters of the amplified product of 260 to 264 bp (intron 8) was digested with 5 to 10 U *Hpa*I for 3 h or at 37 °C overnight. After digestion with *Hpa*I, the products were run on an 8% acrylamide gel with 0.5× Tris-borate-ethylenediaminetetraacetic acid at 200 V for 3 h (Fig. 1). The expected product sizes of different digests are listed in Table 1^[27]^. The differences between both groups (patients and control) were analyzed using SPSS for Windows software (version 18.0). All *p* values were based on two-sided comparisons. *p* values less than 0.05 were considered to indicate statistical significance.

**Table 1:** Intron 8 variation detectable by *Hpa*I restriction digest

**Result of digested products**	**Primers**	
	**CF5T/CF9RR**	**CF7T/CF9RR**
Restriction enzyme	*Hp*aI	*Hpa*I
IVS8-5T allele	237+22(+)	235+22(+)
IVS8-7T allele	261(-)	237+22(+)
IVS8-9T allele	263(-)	261(-)
IVS8 genotype		
*5T/5T*	+/+	-/-
*5T/7T*	+/-	+/+
*5T/9T*	+/-	-/-
*7T/7T*	-/-	+/+
*7T/9T*	-/-	+/-
*9T/9T*	-/-	-/-

Each genotype has a unique *Hpa*I digest pattern when both PCR products are considered.

## RESULTS

**Fig. 2:**
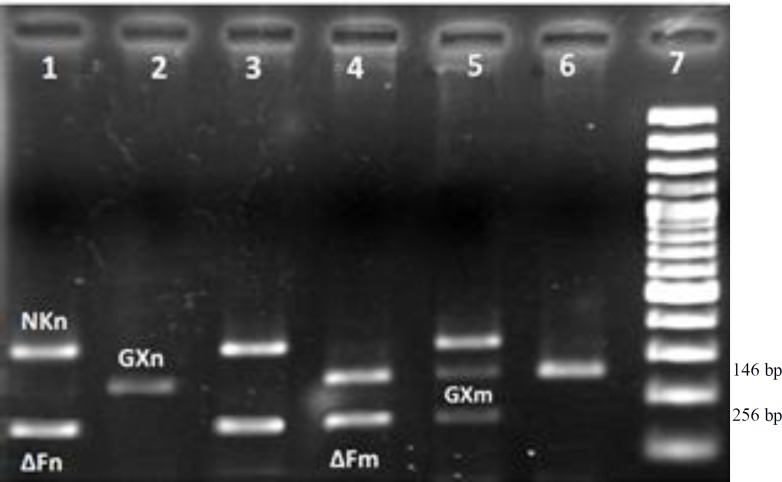
Detection of the most common *CFTR* mutations in CBAVD men by using multiplex ARMS-PCR. Lanes 1 and 2, fertile group (control); lane 3 and 4, heterozygote patients for ∆F508 mutation (∆Fm); Lanes 5 and 6, heterozygote patient for G542X mutation (GXm); Lane 7, 100 pb DNA ladder. NKn, ∆Fn, and GXn represent the PCR products for normal alleles N1303K, ∆F508 and G542X, respectively

Mutation analysis was performed on all the 100 patients (NOA and CBAVD patients) and 100 fertile controls. Among the CBAVD patients, *8/50* (16%), 4/50 (8%), and 4/50 (8%) of *subjects *were heterozygote for F508, G542X, and N1303K, respectively, accounting for 16% of all mutant alleles (Table 2 and Fig. 2).

In the screening of IVS8-Tn polymorphism, seven of the 50 CBAVD patients had the 5T/5T genotype, nine were heterozygous for the 5T/9T genotype, and 11 were heterozygous for the 5T/7T genotype. The frequency of 5T alleles was 34%. The number of patients with genotypes of 9T/9T, 9T/7T, and 7T/7T were 4, 7, and 12, respectively (Table 3). None of the *CFTR* gene mutations were detected in NOA patients, and the frequency of the 5T allele (5%) was similar to the control group. The final results of the IVS8-Tn genotype in NOA and CBVDpatients are presented in Table 3. The frequency of 5T, 7T, and 9T alleles in both groups of the patients and the control are shown in Table 4.

## DISCUSSION

About 97% of men with CF are *infertile* as a result of *CBAVD* and *OA. In addition, *defects in the CFTR* gene may affect the process of spermatogenesis*^[^^19^^,^^20^^,^^23^^]^*. *The frequency of mutations within the *CFTR* gene varies in different populations. F508 is known as the most prevalent mutation in the world^[^^28^^]^. It has also been reported as the most common allele in Iranian CF patients. The other most common mutations are W1282X, G542X, R347H, and R117H^[^^29^^,^^30^^]^. Although numerous investigations have revealed the relationship of *CFTR* gene with infertility problem in populations all over the world, a limited number of such studies have been published in males with CBAVD and other types of infertility in Iran. Hence, the objective of the present study was to investigate the most prevalent mutations in *CFTR* gene in Iranian infertile men (CBAVD and NOA). Selection of the most common mutations (F508, G551D, G542X, N1303K, and W1282X) was based on the high prevalence of these mutations reported in the *CFTR* gene worldwide and also based on the presence of these mutations in patients with CBAVD^[^^19^^,^^20^^]^.

**Table 2:** Frequency of mutations in CBAVD patients

**Mutation**	**Genotype**	**CBAVD patients ** **(n = 16)**	**Genotypic frequencies** **(n = 32)**
G542X	G542X/-	4	8
N1303K	N1303K/-	4	8
F508	F508/-	8	16
W1282X	-/-		
G551D	-/-		

**Table 3:** Final results of IVS8-Tn polymorphism in nonobstructive azoospermia (NOA) and congenital bilateral absence of the vas deferens (CBAVD) patients

**CF5T/CF9RR**	**CF7T/CF9RR**	**IVS8-Tn**	**NOA patients** **(n = 50)**	**CBAVD** ** patients** **(n = 50)**
-/-	+/-	9T/7T	17	7
-/-	+/+	7T/7T	9	12
+/-	-/-	5T/9T	2	9
-/-	-/-	9T/9T	20	4
+/+	-/-	5T/5T	1	7
-/+	+/+	5T/7T	1	11

In our CBAVD patients, F508 with a frequency of 8% was detected as the most common mutation, and then N1303K and G542X (4% and 4%, respectively) were found, which it was *significantly greater than* that of the *control group *(*p* < 0.001). The 5T allele showed a significantly higher rate compared to the control group (34% ver. 3%, *p* < 0.001). These results are consistent with several investigations that evaluated *CFTR* mutations in CBAVD, as well as mutations of F508, 5T, and R117H as the prevalent variations^[^^6^^,^^16^^,^^31^^]^.

In 2007, Radpour *et al.*^[^^32^^]^ published a paper in which different types of mutations were found in the *CFTR* gene among Iranian CBAVD patients. F508 had a high prevalence in patients, and the frequency of IVS8-5T was 27.23%, which *corresponds* to the present investigation (34%). Approximately 35% of Iranian men with CBAVD are carriers for a single mutation or polymorphism in CFTR gene, and around 40% have two mutations (common *CFTR* mutations or IVS8-5T polymorphism)^[^^32^^,^^33^^]^. The frequency of IVS8-5T in our patients was similar to that of Portuguese (27.4%)^[^^34^^]^ and Taiwanese patients (29.2%)^[^^35^^]^ but higher than Turkish (19.6%)^[^^31^^]^.

In our study, the proportion of patients with NOA who had the 5T allele was the same as normal population (5 vs. 3; *p* = 0.721). No mutation was found in this group of patients and healthy normal samples.* The findings of the present study have some similarities and differences *to some previous studies. In the study performed by Heidari *et al.*^[^^32^^]^ for screening two *CFTR* mutations (∆I507 and ∆F508) in Iranian men with NOA, the ΔF508 was found in three individuals; no statistically significant relationship was found between this mutation and NOA^[^^36^^]^. In a recent study, Jiang *et al.*^[^^37^^]^ investigated the prevalence of ∆F508 and R117H mutations, IVS8 poly(T) and TG repeats, in Chinese NOA patients. Their result indicated no mutation in patients and fertile controls, suggesting that these two mutations have a low possibility of being associated with NOA condition. Besides, the T5 allele was identified as the most prevalent factor that increases the risk of having NOA in Chinese. 

In the evaluation of *CFTR* gene mutations in males with various types of *abnormal sperm* parameters by Ślęzak *et al.*^[^^38^^] ^who observed that F508 and IVS8-T occurred in 5.4% of patients, which was similar to the general population. van der Ven *et al.*^[^^21^^]^ have screened 127 males with *poor sperm quality* for 13 *CFTR* gene mutations. The frequency of mutations was significantly higher than the expected CF carrier frequency in the general population, and no mutations were detected in the control group, which was different from our work. In 2011, Safinejad *et al.*^[^^39^^]^ evaluated the common *CFTR* mutations (∆F508, G542X, R117H, W1282X, and N1303K) and the frequency of the 5T variant in men with non-CAVD OA. Their result revealed that 5/53(9.43%) and 4/53(7.55%) of patients were heterozygote for ∆F508 and G542X mutations, respectively, and 5T polymorphism was 14%, which was more frequent than the control subjects (3%; *p* < 0.05). In another research that was carried out on 1195 males with nonobstructive infertility and those with unexplained infertility, no significant difference in *CFTR* gene was identified between the control group and patients^[^^40^^]^. Sharma *et al.*^[^^41^^]^ *have examined* the frequency of *CFTR* mutations in Indian *infertile patients* with NOA and spermatogenic failure. They reported ΔF508 mutation in 3.6% of men with NOA. In this study, a significant correlation was detected between the most common mutations of the *CFTR* gene and IVS8-Tn polymorphism in CBAVD patients. However, the relationship *between this gene *and NOA remains under question. Thus, large-scale cohort studies as well as examination of entire gene or the high number of *CFTR *mutations may be necessary to substantiate the hypothesis of a putative link between a particular combination of *CFTR* mutations and polymorphisms and other types of male infertility.

**Table 4:** Frequency of IVS8-Tn and mutations of *CFTR* gene identified in Iranian NOA and CBAVD patients

**Alleles**	**5T**	**7T**	**9T**	**G542X**	**N1303K**	**F508**	**G551D**	**W1282X**
NOA (n = 50)	5/100	36/100	59/100					
CBAVD (n = 50)	34/100	42/100	24/100	4/100	4/100	8/100	0/100	0/100
Control (n = 100)	3/100	39/100	58/100
